# Intravenous Ferric Carboxymaltose Improves Response to Postoperative Anemia Following Total Knee Arthroplasty: A Prospective Randomized Controlled Trial in Asian Cohort

**DOI:** 10.3390/jcm11092357

**Published:** 2022-04-22

**Authors:** Keun Young Choi, In Jun Koh, Man Soo Kim, Chulkyu Kim, Yong In

**Affiliations:** 1 Department of Orthopaedic Surgery, Seoul St. Mary’s Hospital, College of Medicine, The Catholic University of Korea, Seoul 06591, Korea; heaxagon@hanmail.net (K.Y.C.); kms3779@naver.com (M.S.K.); dreamst00@gmail.com (C.K.); 2 Department of Orthopaedic Surgery, Eunpyeong St. Mary’s Hospital, College of Medicine, The Catholic University of Korea, Seoul 03312, Korea; oskoh74@gmail.com

**Keywords:** total knee arthroplasty, ferric carboxymaltose, Hb responder, iron metabolism, intravenous iron

## Abstract

Background: Ferric carboxymaltose (FCM) is an intravenous (IV) high-dose iron that is effective in the treatment of iron deficiency anemia. This study was performed to determine whether postoperative FCM infusion is effective at improving hemoglobin (Hb) responders, Hb and iron profiles, and the patient’s quality of life (QOL). Methods: A total of 110 patients with postoperative anemia, defined by a Hb < 10 g/dL within 3 days of unilateral primary TKA, between June 2018 and February 2020 were randomized into either the FCM or Control group. On postoperative day 3, the FCM group (55 patients) received IV FCM while the Control group (55 patients) did not. The Hb responders (Hb increase ≥ 2 g/dL compared to baseline), Hb level, iron profiles (ferritin, total iron-binding capacity (TIBC), transferrin saturation (TSAT)), and EQ-5D scores were compared at weeks 2, 4, and 8. Results: The FCM group demonstrated a significantly greater number of Hb responders (*p* < 0.001) and a higher Hb level (*p* = 0.008) at 2 weeks postoperative than did the Control group. The FCM group recovered its preoperative Hb level between 4 and 8 weeks. In contrast, the Control group did not recover its preoperative level until 8 weeks. The FCM infusion group also had higher serum ferritin, iron and TSAT, and lower TIBC levels than those of the Control group between 2 and 8 weeks (all *p* < 0.001). However, there was no significant difference in the postoperative transfusion rate (*p* = 0.741) or EQ-5D score between the two groups (all *p* > 0.05). Discussion: In postoperative anemia following TKA, IV FCM increases the Hb response and improves Hb and iron metabolism variables, however, it does not affect the transfusion rate or QOL.

## 1. Background

Total knee arthroplasty (TKA), the gold standard surgery for advanced knee osteoarthritis (OA), can cause life-threatening postoperative blood loss [1]. The average degree of hemoglobin (Hb) loss due to blood loss following TKA is approximately 3 g/dL [2]. Postoperative anemia is a known independent risk factor for decreased quality of life (QOL), poor physical performance and outcomes, particularly in the elderly, even in the cases which are not so severe to receive allogenic transfusion [3,4]. Theoretically, rapid recovery of the Hb level in patients with mild to moderate anemia could provoke quick recovery and fast rehabilitation, improving the capacity of carrying oxygen in the red blood cells [5]. Bleeding after TKA can also increase the rate of allogenic transfusion, from 3% to up to 69% [6,7]. Allogenic blood transfusion can increase mortality and morbidity of patients, causing an increased length of stay, a higher risk of perioperative infection, increased treatment costs, acute lung injury and sepsis [8]. In order to lower the rate of allogenic transfusion, several options, such as tranexamic acid, an auto-transfusion system and erythropoietin, have been introduced [9,10,11,12,13,14]. In addition, oral or IV iron supplements have been recognized as other options to treat postoperative anemia, because acute bleeding can cause a transient iron-deficient condition. Therefore, iron supplementation may be used as an easy and less (potentially) dangerous way of preventing anemia postoperatively than a blood transfusion [15]. Several prior studies have described the use of iron supplements during various surgeries in various forms. However, to the best of our knowledge, there is no gold standard regimen of iron supplementation that has been shown to lower the risk of postoperative anemia [5,16].

Intravenous (IV) ferric carboxymaltose (FCM) is made of a colloidal solution of nanoparticles that is composed of a polynuclear iron (III)-(oxyhydr) oxide core stabilized by carboxymaltose. IV FCM is a highly concentrated, effective and safe option for rapidly supplementing iron storage and improving anemia in patients with iron deficiency (± anemia) associated with a broad spectrum of etiologies [17,18]. Ferinject^®^ (Vifor Pharma, Flughofstrasse, Switzerland) is a treatment option for patients with iron deficiency when oral iron preparations cannot be used, oral iron preparations are ineffective, or rapid replacement is required [18]. Macrophages take up this complex in the reticuloendothelial system, and it is degraded and transported to the iron transporter protein transferrin in it. This treatment minimizes the amount of ionic iron that is released into the serum. IV FCM complex permits the administration of high doses of iron because of its stability [18]. Previous studies have reviewed the effectiveness of intraoperative IV FCM infusion in TKA and total hip arthroplasty (THA) [19], and in its postoperative use in staged bilateral TKA [20]. However, it is unclear whether postoperative IV FCM infusion is effective for increasing the Hb response in acute postoperative anemia following unilateral TKA and there is also unclear evidence regarding the exact timing of the response. In addition, the relationship between the use of IV FCM and postoperative QOL is unclear.

Therefore, this prospective randomized study was performed to determine whether postoperative administration of IV FCM to patients with postoperative anemia following TKA, especially in the acute period, is effective at: (1) increasing the number of Hb responders; (2) recovering Hb and iron metabolism variable levels; (3) decreasing the transfusion rate and improving QOL. We hypothesized that acute postoperative administration of IV FCM could increase the number of Hb responders in the early postoperative period. We also hypothesize that earlier improvement in Hb and iron metabolism variable levels (compared to those of the control group) would decrease the transfusion rate and increase the QOL.

## 2. Methods

This prospective, single-institution randomized controlled trial was designed as a parallel-group study with balanced randomization. This study protocol was approved by the Institutional Review Board at our hospital. Patients were educated regarding the study requirements, and they provided informed consent. Patients with advanced knee OA who were scheduled for primary unilateral TKA between June 2018 and February 2020 were enrolled in this study. Patients over 20 years old and scheduled to undergo unilateral TKA for primary advanced knee OA were eligible for this clinical trial. As part of the study design, we excluded patients whose Hb level did not decrease to <10 g/dL within 3 days after TKA and those with a history of prior knee surgery affecting bony structures on the ipsilateral knee; and those with chronic liver disease with elevated serum aspartate aminotransferase and alanine aminotransferase more than three times the normal value; known hypersensitivity; or contraindications to iron; anemia treatment within 1 month prior to surgery; severe limitation of motion (a flexion contracture >25 degrees) or who refused to participate in this study. Additionally, for the patients using an anticoagulant agent or antiplatelet agents, proper medical department consultation was done. Patients who could not cease taking those medications or needed heparin bridging were excluded. Patients who could temporarily cease anticoagulants or antiplatelet agents were recommended to stop taking those medications according to medical department recommendations and resumed taking medications after removal of the drain in conditions free from postoperative bleeding. The indicated Hb value for the inclusion criteria was used to assess for moderate anemia [21]. 

**Figure 1:**
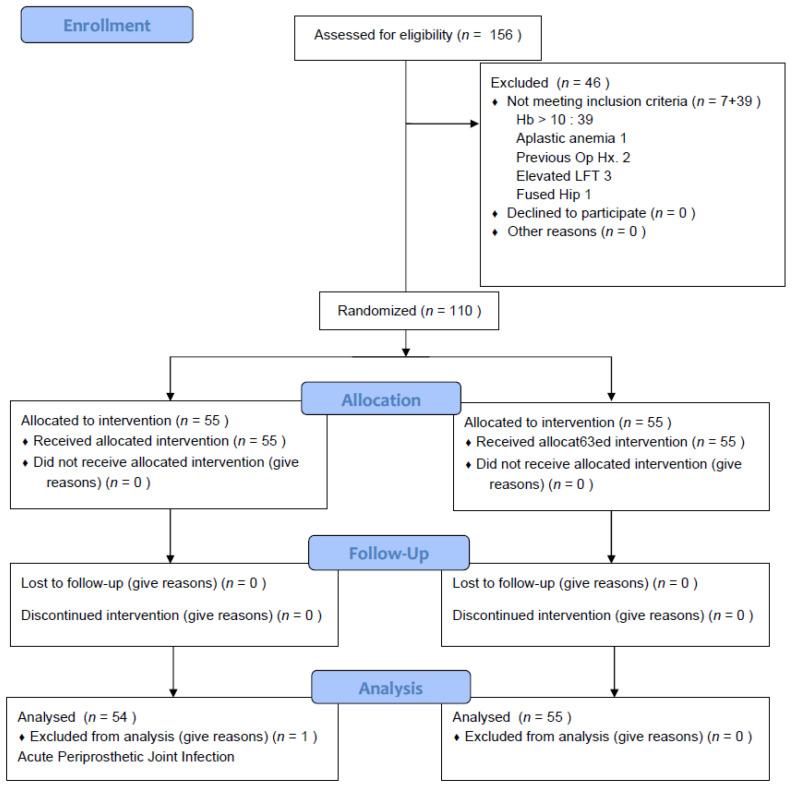
Consolidated Standards of Reporting Trials.

Among the 156 eligible patients, one had aplastic anemia (AA), three had chronic liver disease, one had a fused hip joint affecting the routine operating procedure and two had already undergone operations affecting the bony structures on the ipsilateral knee. Of the 149 patients, 110 (73.8%) fulfilled the inclusion criteria (Hb < 10 g/dL within postoperative 3 days). Thirty-nine patients had a Hb ≥ 10 g/dL for three postoperative days. Eventually, 110 patients were included and randomly assigned to the FCM group (*n* = 55) or the Control group (*n* = 55) by a computer-generated randomization table permuted into blocks of four and six. One investigator (one of the authors) screened the eligible patient and collected the laboratory and clinical data before group allocation on the third postoperative day. Another investigator (one of the authors), who collected the laboratory data after the group allocation, and the other investigator (one of the authors), who collected clinical data associated with QOL were not informed of the group assignments before the completion of the final data analysis. In order to keep the investigators and patients unaware of their prescription, a nurse who was not involved in the patient recruitment administered IV FCM following the order of the investigator who allocated patients into groups. The nurse and the investigator who allocated patients into groups did not give any information to the patient or to the other investigators. In the FCM group, one patient was excluded from the analysis step because of a non-tuberculous mycobacterium infection that required an additional operation at postoperative 8 weeks. Ultimately, 54 patients of the FCM group and 55 patients of the Control group were included in the final analysis step (Figure 1). No significant differences between the groups were found in demographics, other preoperative parameters or surgical parameters (Table 1). 

After group allocation on the third postoperative day, the FCM group received IV FCM, Ferinject^®^ (Vifor Pharma, Flughofstrasse, Switzerland). The Ferinject^®^ infusion was administered, as has been previously described: bodyweight ≥ 50 kg; 1000 mg of Ferinject^®^ mixed with 200 mL normal saline, bodyweight <50 kg; 500 mg of Ferinject^®^ mixed with 100 mL normal saline [18,22]. Patients with a Hb level of <10 g/dL and a serum ferritin level of <15 ng/mL at postoperative 4 weeks were planned to receive an additional dose of 500 mg of FCM [23]. The Hb and iron metabolism variables (ferritin, serum iron, TIBC and TSAT) were assessed one day before surgery and on postoperative weeks 2, 4, and 8. The Hb level was assessed on the day of surgery, as well as the first, second and third postoperative days to evaluate whether the patient was suitable for study inclusion. For the morbidity control, triggers of allogenic transfusions were a Hb of <7.0 g/dL or the symptoms of acute anemia (tachycardia, dizziness, persistent hypotension, chest pain) [24]. In addition, patient self-reported QOL was also assessed on the day prior to surgery and at postoperative weeks 2, 4 and 8 using the EuroQOL-5dimension (EQ-5D)-3L questionnaire. The EQ-5D, a generic measure of health status, was developed by the EuroQol Group (an international research group) [25]. The primary outcome measure was the number of Hb responders at 2 weeks. The Hb responders were defined as patients who accomplished an improvement in Hb levels of 2 g/dL or more, compared to baseline in 2 weeks [26]. The secondary outcome measures included changes in Hb and iron metabolism variable levels, the transfusion rate and patient-reported QOL (as measured by the EQ-5D-3L questionnaire).

All of the surgeries were performed by the senior surgeon (one of the authors) while patients were under general anesthesia. All of the procedures were performed using a subvastus approach with patellar sliding and tourniquet inflation to 300 mmHg. During surgery, 500 mg IV tranexamic acid was administrated before the tourniquet was released. In all cases, intra-articular suction catheters were placed and removed within 48 h postoperatively. Antithrombotic treatment was initiated on the first postoperative day. All of the patients received subcutaneous Enoxaparin (0.2 mL 2000 IU; Clexane, Sanofi-Aventis, France) for 5 days at 24-h intervals. Other routine medical treatments that could affect blood loss or coagulation pathways were not applied. Joint aspiration for postoperative hemarthrosis after removal of the drain catheter was not done in every participant. On the day of surgery, the patients were encouraged to extend their knees every one hour, to stay in bed to prevent falls, but not to limit ambulation entirely. On the first postoperative day, the patients were encouraged to walk with a walker and engage in increasingly strenuous range-of-motion exercises while they were in bed. The patients were discharged one week after surgery. Follow-up visits were scheduled for 2, 4 and 8 weeks postoperatively, as well as 3 and 6 months and 1 year postoperatively. They were followed annually after this interval.

### Statistical Methods

We used the average results of previous studies to determine the sufficient sample size required for statistical power [23]. Calculations were based on a superiority design assuming an FCM response of 83.5% by postoperative 2 weeks and a response of 57% in the Control group by postoperative 2 weeks. Based on these estimates, 43 patients were required to discriminate a significant difference at the 5% level with 80% power. At least 96 patients (48 in each group) are required regarding a 10% of drop rate. The primary and secondary endpoints were compared between the two groups. The chi-square test was used to determine differences in the categorical variables, including the number of Hb responders and the transfusion rate. The Student’s *t*-test was used to analyze continuous variables, including Hb, iron metabolism variable levels and the EQ-5D-3L. The paired t-test was used to analyze differences in the Hb and iron metabolism variable levels at some postoperative moment compared to a preoperative one. All of the computations were performed using standard software (SPSS 21.0; SPSS Inc, Chicago, IL, USA) with a significance set at *p* < 0.05. Multiple comparisons were performed with repeated-measures analysis of variance followed by the Bonferroni corrected post hoc analysis.

## 3. Results

**Figure 2:**
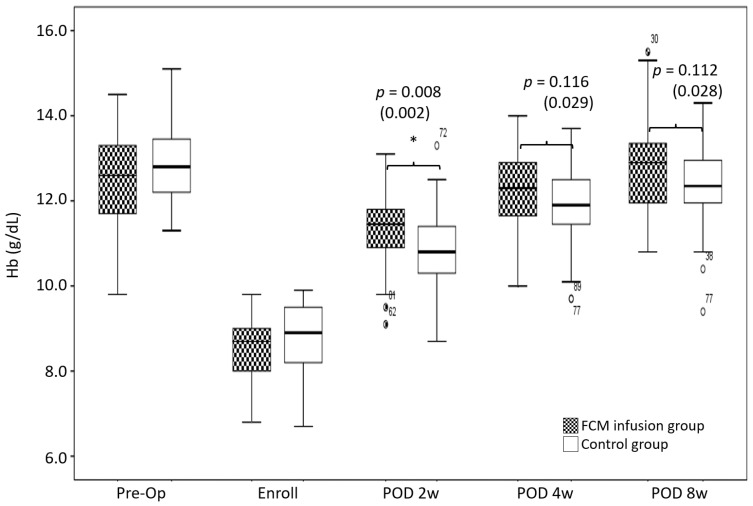
Comparisons of the Hb level between the FCM and Control groups. The asterisks demonstrate a significant difference between groups. Band within the box = median; top and bottom of box = interquartile range (IQR); whiskers = values within 1.5 IQR of the top or bottom of the box; black and white circles with number = outliers in FCM group with case number indicating the case number; and white circles with number = outliers in Control group with case number indicating the case number. The *p*-values corrected by Bonferroni analysis are indicated, with the *p*-values before the Bonferroni analysis in parentheses. Hb: hemoglobin; FCM: ferric carboxymaltose; Pre-op: preoperative; w: week.

The number of Hb responders at two weeks postoperatively was greater in the FCM group than it was in the Control group (88.9% in the FCM group vs 49.1% in the Control group, *p* < 0.001) (Table 2). The FCM group had a higher Hb level than did the Control group at 2 weeks (*p* = 0.008) (Figure 2). The FCM group recovered to the preoperative Hb level between 4 and 8 weeks (Hb (preop); 12.5 ± 1.0 g/dL, Hb (2 W); 11.4 ± 0.8 g/dL, Hb (4 W); 12.2 ± 0.9 g/dL, and Hb (8 W); 12.8 ± 1.1 g/dL) (preop vs. 2 W; *p* < 0.001, vs. 4 W; *p* = 0.026, vs. 8 W; *p* = 0.016). In contrast, the Control group did not recover its preoperative Hb level until 8 weeks (Hb (preop); 12.8 ± 0.8 g/dL, Hb (2 W); 10.9 ± 0.9 g/dL, Hb (4 W); 11.9 ± 0.9 g/dL and Hb (8 W); 12.4 ± 0.9 g/dL) (preop vs. 2, 4 and 8 W; *p* < 0.001) (Figure 2).

**Figure 3:**
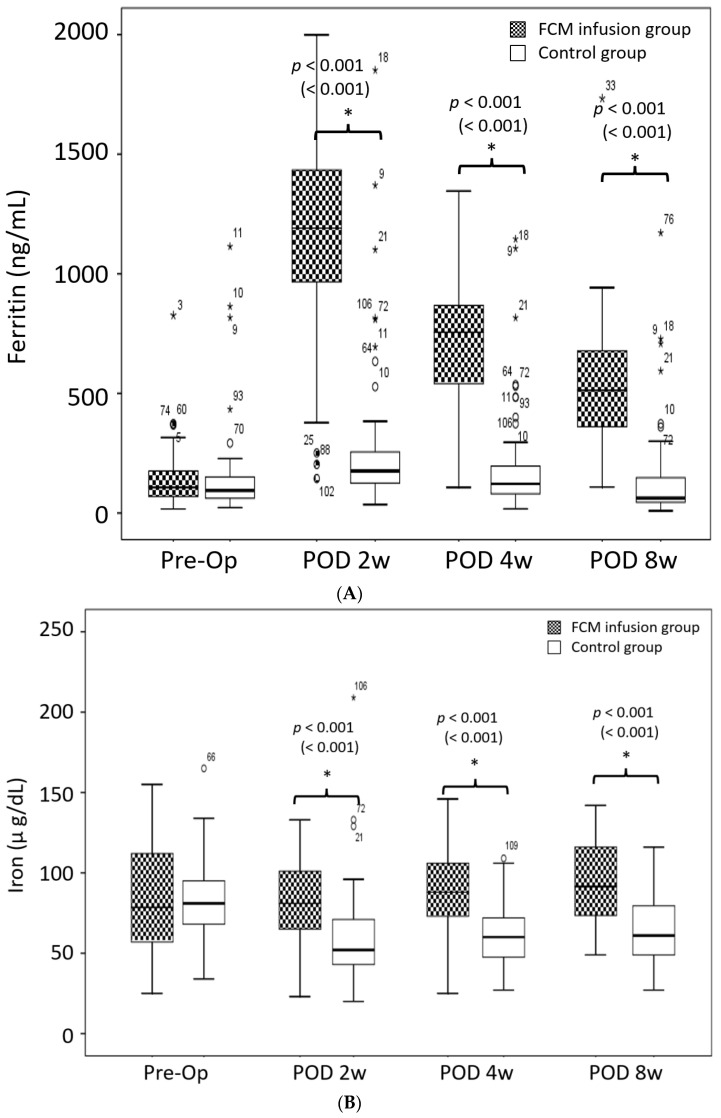

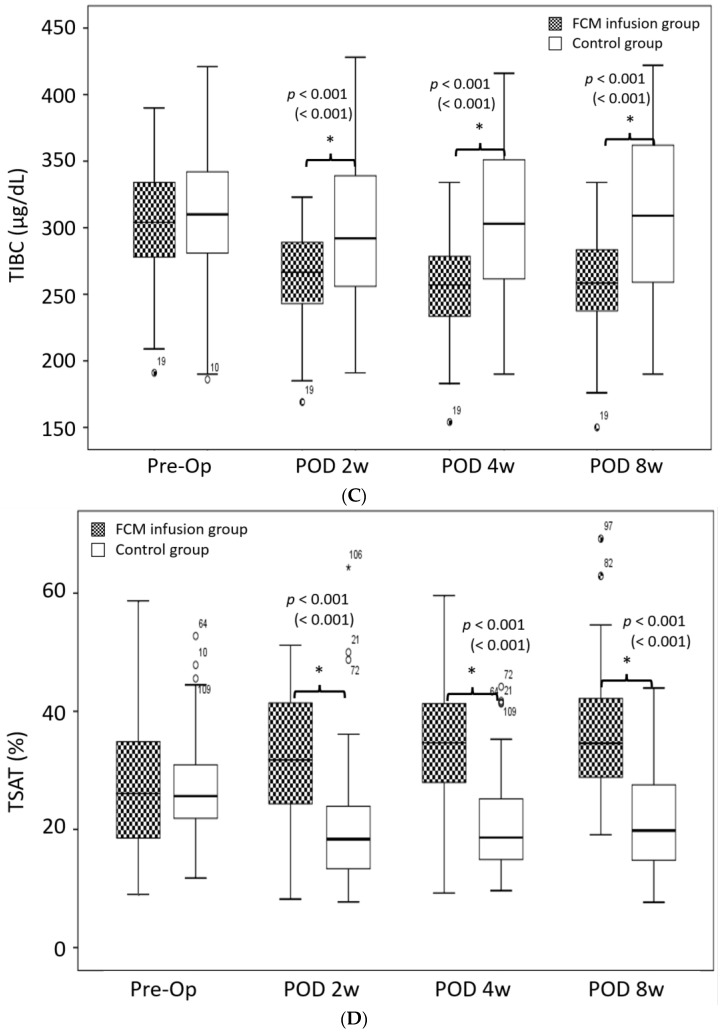
(**A**). Comparisons of the Ferritin level between the FCM and Control groups. (**B**). Comparisons of the serum iron level between the FCM and Control groups. (**C**). Comparisons of the TIBC level between the FCM and Control groups. (**D**). Comparisons of the TSAT level between the FCM and Control groups. The asterisks demonstrate a significant difference between groups. Band within the box = median; top and bottom of box = interquartile range (IQR); whiskers = values within 1.5 IQR of the top or bottom of the box; black and white circles with number = outliers in FCM group with case number indicating the case number; white circles with number = outliers in Control group with case number indicating the case number; and star = extreme outlier in each group. The *p*-values corrected by Bonferroni analysis are indicated, with the *p*-values before the Bonferroni analysis in parentheses. FCM: Ferric carboxymaltose; Pre-op: Preoperative; POD: Postoperative day; w: week; TIBC: total iron binding capacity; TSAT: transferrin saturation.

The FCM group showed better postoperative iron metabolism variables than did the Control group from postoperative 2 weeks to 8 weeks (all *p* < 0.001) (Figure 3). There was a higher ferritin level in the FCM group from 2 to 8 weeks from that at baseline (all *p* < 0.001). However, in the Control group, the ferritin level surpassed the preoperative level only at 2 weeks postoperatively (preop vs. 2 W; *p* = 0.003, vs. 4 W; *p* = 0.132, vs. 8 W; *p* = 0.748) (Figure 3A). The serum iron level in the FCM group was maintained from baseline at 4 postoperatively; however, it surpassed the preoperative level at 8 weeks postoperatively (preop vs. 2 W; *p* = 0.920, vs. 4 W; *p* = 0.575, vs. 8 W; *p* = 0.023). However, in the Control group, the serum iron level decreased below the preoperative level and did not recover until 8 weeks postoperatively (preop vs. 2, 4 and 8 W; all *p* < 0.001) (Figure 3B). The FCM group maintained a low TIBC level until 8 weeks postoperatively (preop vs. 2, 4 and 8 W; all *p* < 0.001). In contrast, the TIBC level in the control group decreased below the preoperative level at 2 weeks postoperatively and then recovered to the preoperative level at 4 weeks (preop vs. 2 W; *p* = 0.018, vs. 4 W; *p* = 0.778, vs. 8 W; *p* = 0.251) (Figure 3C). The FCM group demonstrated a higher TSAT level from 2 to 8 weeks (preop vs. 2, 4 and 8 W; all *p* < 0.05) postoperatively compared to the baseline level. However, in the Control group, the TSAT level decreased below the preoperative level from 2 to 8 weeks postoperatively (preop vs. 2, 4 and 8 W; *p* < 0.001) (Figure 3D).

Postoperatively, seven (13.0%) patients in the FCM group and six (10.9%) in the Control group were treated with allogenic transfusions. There was no difference in the transfusion rate between the groups (*p* = 0.741). Although seven patients in the FCM infusion group and six in the Control group received allogenic transfusions, only one patient in each group met the laboratory criteria (Hb < 7 g/dL) for these. The transfusions in the other cases were administered in the treatment of symptomatic anemia. Moreover, there were no significant differences between the groups in the EQ-5D scores throughout the follow-up period (all *p* > 0.05) (Table 3). There were no FCM infusion-related adverse events or iron-related adverse events, such as headache, pruritus or phlebitis in the FCM infusion group and there also were no other complications that were independent of IV FCM, such as wound complications or serious complications that require additional treatment in both groups during the follow-up period (*p* > 0.05).

## 4. Discussion

The most important finding of this study is that a single postoperative infusion of IV FCM improved the Hb response in acute postoperative anemia. Postoperative anemia following TKA is a known risk factor for poor physical performance and QOL, even in mild to moderate cases [3,4]. Quick recovery of the Hb level in such patients encourages fast recovery and early rehabilitation, as it increases the oxygen-carrying capacity of the red blood cells in the tissues [5]. Recent studies reported that IV FCM is associated with early Hb response after other surgical and medical departments [22,23]. However, the effect of IV FCM on patients undergoing TKA is unclear. Therefore, we evaluated whether a modification to the blood managing protocol, including IV FCM, is necessary to facilitate rapid recovery in terms of objective and subjective outcome measurements in TKA.

Our study results support the hypothesis that the postoperative use of IV FCM could increase the number of Hb responders. In this study, a single infusion of either 500 mg or 1000 mg of IV FCM increased the number of Hb responders at postoperative 2 weeks (88.9% of patients in the FCM group vs. 49.1% of patients in the Control group) (*p* < 0.001) (Table 2). Our results do not concur with a previous study of staged bilateral TKA, which showed no differences in the Hb levels between patients with and without postoperative IV iron supplementation [20]. The reason for this discordance is not clear, but it might be caused by different protocols and situations across the two studies. The patients in our study underwent a single surgical procedure within at least 3 months. In contrast, those in the other study underwent their second TKA one week after the IV iron injection. Based on these differences, patients must be given adequate time for erythropoiesis and iron utilization after IV iron supplementation. Our results suggest that in order to derive the most benefit from IV FCM infusion, more than 2 weeks are needed. Therefore, additional surgery during this period is not recommended. 

With regard to the postoperative Hb levels, this study supports the hypothesis that the postoperative use of IV FCM could increase the Hb level in the acute postoperative period. Our results revealed a significant increase in the serum Hb level at 2 weeks (2 w; 11.4 ± 0.8 g/dL in IV FCM group vs. 10.9 ± 0.9 g/dL in Control group) (*p* = 0.008) (Figure 2). In addition, patients in the FCM group recovered to their preoperative Hb levels faster than did those in the Control group (between 4 to 8 weeks in the FCM group vs. not until 8 weeks in the Control group). However, the findings of this study contradict prior suggestions that IV FCM does not influence the postoperative Hb level [20]. In addition to the study’s differences previously mentioned, this disagreement may be explained by different blood management protocols. Because of the different study designs and the low adverse event rate of IV FCM in this study, we recommend the use of IV FCM as a blood managing protocol after unilateral TKA. Our study results support the hypothesis that postoperative IV FCM can improve iron metabolism variable levels. The FCM group had higher ferritin, serum iron and TSAT levels, but lower TIBC levels at postoperative weeks 2, 4 and 8 compared to those at baseline (all *p* < 0.001) (Figure 3). These results are consistent with prior work showing a faster recovery and higher iron metabolism levels in patients receiving iron supplementation [20,23]. The IV form of iron provides high utilization of administered iron and is well tolerated. These properties allow it to be administered at high, single doses [27]. None of the patients in the FCM infusion group met the additional infusion criteria at postoperative 4 weeks. These patients also had improved iron metabolism variable levels, which were maintained throughout the follow-up period. Therefore, a single infusion of FCM may eliminate the need for oral or IV iron supplements for postoperative anemia.

Despite improvements in the Hb response and iron metabolism variables with IV FCM, our results could not support the hypothesis that these improvements would reduce the transfusion rate or improve patient report QOL. The transfusion rate between the two groups showed no difference (*n* = 7, 13.0% in the FCM group vs. *n* = 6, 10.9% in the Control group). These findings concur with previous reports that iron supplements do not lower the transfusion rate after TKA [5,20]. Therefore, our data and previous data suggest that IV FCM does not influence the transfusion rate following TKA. However, only one patient in each group required an allogenic transfusion because of a low Hb level. In contrast, the other transfusions (six in the FCM infusion group, and five in the Control group) were administered for clinical symptoms. Still, larger studies are needed to accurately analyze the impact of postoperative FCM infusions on the transfusion rate. Similarly, IV FCM did not influence the EQ-5D score (all *p* > 0.05) (Table 3). The results of this study concur with previous studies in other surgical departments, which found that better iron metabolism profiles do not directly indicate better QOL [23]. One previous study found that the improvements in EQ-5D scores mostly occurred between 6 weeks to 3 months postoperatively after TKA. In contrast, we found that there was no difference in the Hb level from 4 weeks after TKA. The effect of IV FCM on a patient’s QOL is obscure [28]. 

In addition, although we did not compare cost-effectiveness as a secondary outcome measure, IV FCM did not cause a remarkable variation in total in-hospital stay costs. Korea has a compulsory national health insurance system with universal coverage, and the Health Insurance Review and Assessment service, a public agency sponsored by the Ministry of Health and Welfare, review the costs of health care benefits and evaluates the reasonableness of health care services provided by medical institutions. We calculated the cost based on this system. Generally, unilateral TKA costs about USD $2500 on the assumption that the patient stays in a hospital for one week in a Korean training hospital. IV FCM costs about USD $130 for one vial (one pint of PCR costs about USD $71). Calculating the total cost, if IV FCM is applied, it represents about 5.2% of the total in-hospital stay cost for each vial, and the transfusion represents about 2.8% for each pint. Although IV FCM could not completely replace the transfusion, regarding the low adverse event rate of IV FCM in this study, IV FCM could be used as a relatively safe and cost-effective tool for recovering Hb levels. 

There are several limitations to this study. First, the population included in this study is confined to Asians. Therefore, our results may not be generalizable to other populations. Most patients who underwent unilateral TKA included in this study were female (97/110, 88.2%). Although the reason is not clear, the majority of patients with advanced knee OA in Korea are females [29]. A second limitation is that this study was confined to unilateral TKA. Additional studies are needed to determine whether there is any difference in the Hb response, Hb and iron metabolism variables level, transfusion rate and QOL after a second operation or the same-day bilateral TKA. A third limitation is that the sample size of this study was estimated to test our primary outcome. There is a possibility that this study was underpowered and subject to type-II error in terms of detecting all relevant outcomes. Fourth, a placebo is not included as a counterpart of IV FCM. Therefore, the degree of blinding was not as strict as that in a placebo-controlled trial. Fifth, although a variety of assessment tools and scales could be used to assess the patient’s QOL after TKA, we choose the EQ-5D-3L. However, other scales may be necessary for a more accurate evaluation of QOL. Because the EQ-5D-5L discriminates more strongly than EQ-5D-3L did, we believe that it is better to use the EQ-5D-5L than the EQ-5D-3L in further studies [30]. Sixth, the patient’s preoperative status of iron storage can influence the improvement of postoperative anemia. Although the patients in both groups had similar preoperative demographics and iron metabolism profiles, there may be hidden causes that affect iron storage (such as oral intake and lifestyle). Seventh, the perioperative blood managing strategy, which may differ between studies, could have affected the study result. The obstacles to this study’s generalizability include the diversity of dosage and timing of IV FCM applications, the trigger points of transfusions and the postoperative antithrombic regimens. An eighth limitation is that the patients’ chronic medications, such as antithrombic and antiplatelet agents, may have influenced their bleeding tendency and the study results. Ninth, the use of a tourniquet and drain might have affected the study result. Although there are several studies that a tourniquet and drain do not affect total blood loss [31,32,33], there are also few studies that reported that a tourniquet and drain can increase blood loss [34]. Additionally, there are diverse results for drain clamping [35,36,37]. Regarding these various study results, interpretation of our study results requires careful consideration. Despite these limitations, this randomized clinical trial provides worthy information regarding the effects of IV FCM on the Hb response, Hb and iron metabolism variables, transfusion rates and QOL in the acute postoperative period after unilateral TKA.

In conclusion, a single postoperative infusion of IV FCM increased the Hb response and improved Hb and iron metabolism levels in acute postoperative anemia, but did not affect the transfusion rate or the patient’s QOL.

## Figures and Tables

**Table 1:** Patient Demographics and Preoperative Characteristics.

Demographics	FCM (*n* = 54)	Control (*n* = 55)	*p*-Value ‡
Age * (year)	71.4 (5.7)	71.8 (6.2)	0.768
Female Sex †	50 (92.6)	47 (85.5)	0.234
BMI * (kg/m^2^)	26.4 (4.6)	25.6 (3.4)	0.273
ASA status †			0.715
I, II	51 (94.4)	51 (92.7)	
≥III	3 (5.6)	4 (7.3)	
Tourniquet time * (min)	42.9 (6.8)	41.4 (8.0)	0.306
Operation time * (min)	74.1 (11.1)	76.2 (11.1)	0.333
Total Hemovac * (mL)	484.5 (278.1)	449.6 (271.9)	0.510
Comorbidities †			
HTN	32 (59.3)	34 (61.8)	0.785
DM	19 (35.2)	15 (27.3)	0.373
Brain	3 (5.6)	4 (7.3)	0.715
Thyroid	6 (11.1)	5 (9.1)	0.726
Kidney	2 (3.7)	5 (9.1)	0.251
Lung	4 (7.4)	8 (14.5)	0.234
Liver	1 (1.9)	3 (5.5)	0.317
Smoking †	0 (0.0)	2 (3.6)	0.157
Alcohol †	4 (7.4)	3 (5.5)	0.678
CRP * (mg/dL)	0.18 (0.31)	0.17 (0.42)	1.000 (0.880)
CrCl * (mg/dL)	76.1 (18.0)	81.4 (17.3)	0.496 (0.124)

FCM: ferric carboxymaltose; BMI: body mass index; ASA: american society of anesthesiologists; DM: diabetes mellitus; CRP: c-reactive protein; CrCL: creatine clearance. * Data are presented as means (standard deviations). † Data are presented as numbers (percentage) of patients. ‡ The *p*-values corrected by Bonferroni analysis are presented, with the *p*-values before the Bonferroni analysis in parentheses.

**Table 2:** Postoperative Hb responders.

Hb Responder †	FCM (*n* = 54)	Control (*n* = 55)	*p*-Value ‡
POD 2 w	49 (88.9)	27 (49.1)	<0.001 (<0.001)
POD 4 w	53 (95.9)	49 (90.0)	1.000 (0.251)
POD 8 w	54 (97.9)	53 (98.1)	1.000 (0.954)

Hb: hemoglobin; FCM: ferric carboxymaltose; POD: postoperative day; w: week. † Data are presented as numbers (percentage) of patients. ‡ The *p*-values corrected by Bonferroni analysis are presented, with the *p*-values before the Bonferroni analysis in parentheses.

**Table 3:** Postoperative patient report QOL (EQ5D-3L).

EQ-5D Total *	FCM (*n* = 54)	Control (*n* = 55)	*p*-Value ‡
Preop	8.9 (1.7)	8.8 (1.4)	1.000 (0.861)
2 w	9.4 (1.2)	9.5 (1.6)	1.000 (0.752)
4 w	8.9 (1.3)	8.9 (1.5)	1.000 (0.992)
8 w	8.1 (1.5)	8.1 (1.5)	1.000 (0.955)

QOL: quality of life; EQ-5D: EuroQOL-5dimension; FCM: ferric carboxymaltose; Preop: preoperative; w: week. * Data are presented as the means (standard deviations). ‡ The *p*-values corrected by Bonferroni analysis are presented, with the *p*-values before the Bonferroni analysis in parentheses.

## Data Availability

Data collected for this study, including individual patient data, will not be made available.

## Abbreviations

FCM	Ferric Carboxymaltose
IV	Intravenous
Hb	Hemoglobin
QOL	Quality of life
TIBC	Total iron binding capacity
TSAT	Transferrin saturation
TKA	Total knee arthroplasty
OA	Osteoarthritis
THA	Total hip arthroplasty
AA	Aplastic anemia
Preop	Preoperative
